# Gender differences in symptoms suggestive of diabetic gastroparesis in the West Bank: clinical insights from a cross-sectional study

**DOI:** 10.3389/fmed.2026.1735327

**Published:** 2026-06-10

**Authors:** Diya Asad, Qusai Zreqat, Shahd T. Idais, Marah Hunjul, Bara'ah Huseein, Alaa Ayyad, Hamzeh M. I. Abugharbieh, Haroun Neiroukh, Areen Zuhour, Salsabeel M. Abukhalaf, Nour Al-Atrash, Roa Alzughayyar, Hussein Hallak

**Affiliations:** 1 Faculty of Medicine, Al-Quds University, Jerusalem, Palestine; 2 School of Medicine, Faculty of Medicine and Health Sciences, An-Najah National University, Nablus, Palestine

**Keywords:** Gastroparesis Cardinal Symptom Index (GCSI), gender differences, symptoms suggestive of diabetic gastroparesis, type 2 diabetes mellitus, West Bank - Palestine

## Abstract

**Introduction:**

Diabetic gastroparesis (DGP) is a common complication among DM patients, with a prevalence between 5 and 15%, manifest as upper gastrointestinal symptoms, including nausea, postprandial fullness, vomiting, early satiety, and bloating. The pathogenesis is not completely understood, and has been linked with multiple risk factors, one of which is the female gender. Although females are 4 times more likely to develop DGP, there is still not enough evidence on the exact mechanisms underlying sex-related differences. In this research, we aim to conduct a cross-sectional survey to provide large-scale, region-specific data on gender differences in both the prevalence and severity of gastroparesis symptoms, as well as to identify independent associated factors of symptoms burden among patients with type 2 DM.

**Methods:**

A cross-sectional study was conducted, a total of 3,248 were included in our study, and assessed gastroparesis symptoms using a validated Arabic version of the Gastroparesis Cardinal Symptom Index (GCSI).

**Results:**

Our study showed that females had a higher prevalence of obesity, poorer glycemic control, and DGP and were more likely to have a severe picture of DGP related symptoms (GCSI score of ≥3.5). Female patients reported a significantly higher prevalence of all gastroparesis symptoms compared to male patients, and reported significantly higher mean severity scores for all symptoms. Logistic regression also showed that female gender is the only independent associated factor with the severe form of gastroparesis symptoms compared to male (OR: 2.38) *p* value (0.019), while other variables were not significantly associated.

**Discussion:**

DGP prevalence is 4 times higher in females and reported higher severity scores of DGP symptoms, especially nausea, early satiety, fullness, bloating, and loss of appetite. Multiple theories have tried to explain, including slower gastric emptying in females due to estrogen hormone and neuronal nitric oxide synthase, which affects GI smooth muscle motility and some physiological differences.

**Conclusion:**

Female gender is an associated factor for developing DGP and the only independent factor associated with DGP severity. However, the underlying mechanism is not fully understood. This raises the need for targeted research, as a better understanding of DGP is a necessity to implement gender-based management plans.

## Introduction

Diabetic gastroparesis (DGP) is a medical term that refers to upper gastrointestinal symptoms in patients with diabetes mellitus (DM) due to abnormal gastric emptying (1), it typically manifests as nausea, postprandial fullness, vomiting, early satiety, and bloating (2), DGP affects patients with long-standing diabetes mellitus (DM), with a prevalence between 5 and 15% (1, 3), although it is more commonly associated with long disease duration, cases have also been reported in adolescents with poor glycemic control, suggesting that symptom onset may occur earlier in the disease course (4). Once the patient develops gastroparesis, symptoms may persist for 12–25 years despite glycemic control (5), therefore DGP is associated with worse well-being and increased healthcare burden morbidity and mortality around the world (5, 6).

The gold standard to diagnose DGP is scintigraphy (5), however, this method requires special equipment and exposes patients to radiation (7), Gastroparesis Cardinal Symptom Index (GCSI), has been utilized as a screening tool for DGP related symptoms, as it assesses the severity of symptoms associated with gastroparesis, including nausea, bloating, and postprandial fullness. The GCSI has demonstrated an internal consistency of 0.84 and a test–retest reliability of 0.76 (8), making it a valuable screening tool in countries with resource-limited settings lacking access to scintigraphy.

DGP’s true pathogenesis is not completely understood, but it has been linked with poor glycemic control, neuromuscular dysfunction, sympathetic and parasympathetic neuropathy, and hormonal imbalance (1, 5). Autonomic neuronal damage is thought to reduce the frequency of gastric muscle contractions, ultimately delaying gastric emptying (9).

Multiple risk factors for DGP were established, such as gender, gastric and diabetic surgery, diseases involving the connective tissue and the nervous system, and the use of certain drugs (10). Female gender is considered an associated factor for DGP, Where symptoms related to gastroparesis are more common in females compared to males with type 2 diabetes mellitus (T2DM) (11) this could be attributed to slower gastric emptying in females due to estrogen hormone and neuronal nitric oxide synthase that affects smooth muscle motility (11, 12) also a higher level of estrogen and progesterone in the luteal phase were associated with decreased gastric motility (13). However, the exact mechanisms underlying sex-related differences remain unclear.

Although sex-related differences in diabetic gastroparesis have been reported in previous studies, population-based data from the Middle East remain scarce. Furthermore, most available studies are limited by small sample sizes or are conducted in settings with access to advanced diagnostic modalities. In resource-limited settings such as Palestine, symptom-based tools like the Gastroparesis Cardinal Symptom Index (GCSI) are often the primary method of assessment. Therefore, this study aims to provide large-scale, region-specific data on gender differences in both the prevalence and severity of gastroparesis symptoms, as well as to identify independent associated factors with symptom burden among patients with type 2 diabetes mellitus.

## Methodology

### Study design and study population

This was a cross-sectional study conducted between February 2020 and January 2022 across the West Bank, Palestine. A total of seven governmental hospitals were included, each has a capacity exceeding 100 beds.

The target population comprised patients with type 2 diabetes mellitus (T2DM) residing in Palestine. A total of 3,542 T2DM patients were approached for participation.

### Inclusion and exclusion criteria

Inclusion criteria were all T2DM patients aged 18 years or older., we excluded any patients with a history of medical conditions that could independently cause gastroparesis related symptoms, such as gastrointestinal (GI) disorders, liver disease, collagen vascular disorders, thyroid disease, Parkinson’s disease, or chronic renal disease, patients who underwent gastrointestinal surgeries, or use of any medications that affect gastric motility such as opiates, anticholinergics, tetrahydrocannabinol (THC). Patients who declined participation or did not complete the survey were also excluded.

### Data collection and data sampling

Data collection was performed through face-to-face interviews using a structured questionnaire using the KoboToolbox mobile data collection platform on smartphone devices. The questionnaire included demographic variables (age, sex, weight, height), clinical variables (duration of diabetes, treatment modalities), metabolic parameters (fasting blood glucose and HbA1c levels as reported from recent medical records), and lifestyle factors (dietary adherence and physical activity). Body mass index (BMI) was calculated as weight in kilograms divided by height in meters squared, and obesity was defined as BMI ≥ 30 kg/m^2^. Glycemic control was assessed based on the most recent HbA1c value. Participants were selected through simple random sampling.

### Sample size

The Palestinian Central Bureau of Statistics (PCBS) showed that the population of the West Bank in 2019 was approximately 2.99 million (1.53 million males and 1.46 million females). Based on a T2DM prevalence of 15.3%, and the prevalence of diabetic gastroparesis-related symptoms is estimated to range from 5 to 12%, the sample size was calculated using an online calculator with a 95% confidence level and a 5% margin of error.

### Symptoms assessment

The gastroparesis symptoms were assessed using a validated Arabic version of the gastroparesis Cardinal Symptom Index (GCSI) (3, 14). This tool includes nine items derived from the PAGI-SYM instrument, assessing symptom severity on a 5-point Likert scale. In recent studies, a GCSI score of ≥1.90 has been considered indicative of clinically significant gastroparesis symptoms (15, 16).

### The questionnaire components

The structured questionnaire consisted of three main parts: the first covers participant sociodemographic characteristics such as age, gender, weight, and height. The second covers clinical data, including T2DM history and management, including duration of T2DM, Last fasting blood sugar, Last HbA1c, glycemic control status, and control plan (diet, medication, or both). The third part was the Arabic version of GCSI.

### Data analysis

Data were analyzed using the Statistical Package for Social Sciences (SPSS) version 25. Descriptive statistics were used to summarize patient characteristics, categorical variables were presented as frequencies and percentages, whereas continuous variables were presented as means and standard deviations.

An independent T-sample test was used to assess the cross-gender differences in Age, HbA1c, FBS, BMI, and DM duration, and it was used to compare severity scores of symptoms suggestive of gastroparesis.

Chi-square tests and Fisher’s Exact Tests were used to assess associations between categorical variables and gender, and to evaluate the prevalence of diabetic gastroparesis symptoms across gender.

A binary logistic regression model was developed to evaluate the association between gender, HbA1c, age, and the presence of gastroparesis symptoms. Odds ratios (ORs) were reported along with 95% confidence intervals (CIs). All tests were two-sided, and a *p*-value < 0.05 was considered statistically significant.

### Ethical considerations

This study was carried out in accordance with the principles of the Declaration of Helsinki, following approval from the Research Ethics Committee of Al-Quds University (Ref No: 181/REC/2021). Informed consent was obtained from all participants prior to the start of the study.”

## Results

This cross-sectional study included 3,542 patients with type 2 diabetes mellitus (T2DM); only 3.248 (91.6%) were included in the analysis, comprising 1,927 (59.3%) females and 1,321 (40.7%) males. Table 1 outlines the clinical characteristics of the participants. Female patients showed a higher prevalence of obesity, poorer glycemic control, and DGP (GCSI score of ≥1.90) and were more likely to have a severe picture of DGP (GCSI score of ≥3.5). However, no statistically significant differences were observed between males and females regarding self-reported adherence to dietary counseling or physical activity.

**Table 1:** Disease characteristics of T2DM patients compared by gender.

Demographic Data	Females *N* = 1927	Males *N* = 1,321	*p* values**
Age(years)*	61 (±10)	62 (±11)	0.122
BMI (kg/cm^2^)*	32.1 (±12.7)	29.7 (±9.9)	0.001
Obesity (%)	935 (48.5%)	503 (38%)	0.001
Diabetes duration (years)*	11 (±8)	13 (±9)	0.001
<5 years	413 (21.4%)	248 (18.8%)	
5–10 years	669 (34.7%)	406 (30.7%)	
more than 10 years	845 (43.9%)	667 (50.5%)	
Fasting blood sugar (mg/dl)*	189 (±82)	187 (±83)	0.575
HbA1c (%)	8.27 (±1.95)	8.49 (±2.02)	0.003
Medications			
Metformin use (%)	1,459 (86.2%)	965 (77.3%)	0.001
Insulin use (%)	624 (32.4%)	508 (38.5%)	0.001
Lifestyle			
Exercise and sport (%)	13 (0.7%)	15 (1.1%)	0.163
Diabetes diet (%)	950 (49.3%)	604 (45.7%)	0.045
Diabetic gatroparesis	325 (16.9%)	145 (11%)	0.001
Severe DGP	36 (1.9%)	12 (0.9%)	0.026

Values are expressed in percentages. *Mean ± standard deviation; Obesity = BMI ≥ 30 kg/cm^2^. **p value is for a cross-group comparison; *post hoc* testing was conducted for significant findings.

To determine gender differences in the prevalence and severity of gastroparesis symptoms, results from Tables 2, 3 were analyzed. Female patients reported a significantly higher prevalence of all gastroparesis symptoms compared to male patients; (nausea (*p* = 0.001), retching (*p* = 0.001), vomiting (*p* = 0.025), stomach fullness (*p* = 0.001), not being able to finish a normal-sized meal (*p* = 0.001), feeling excessively full after meals (*p* = 0.001), and loss of appetite (*p* = 0.001)). Additionally, females reported significantly higher mean severity scores for all symptoms.

**Table 2:** Prevalence of symptoms suggestive of gastroparesis compared by gender (%).

Symptomes	Females *N* = 1927	Males *N* = 1,321	*p* value**
Nausea (%)	43.2%	33.3%	0.001
Retching (%)	33.4%	24.9%	0.001
Vomiting (%)	18.7%	15.7%	0.025
Stomach fullness (%)	50.3%	42.7%	0.001
Early satiety (%)	48.9%	38.8%	0.001
Feeling excessively full after meals (%)	43.4%	35.3%	0.001
Loss of appetite (%)	43%	35.8%	0.001
Bloating (%)	50.5%	41.1%	0.001
Stomach or belly visibly larger (%)	36.4%	28.7%	0.001
Any symptom (%)	83.2%	75.9%	0.001
No. of symptoms*	3.68 ± 2.67	2.96 ± 2.65	0.001

*Mean ± standard deviation. **p value is for a cross-group comparison; *post hoc* testing was conducted for significant findings.

**Table 3:** Mean severity score of symptoms suggestive of gastroparesis compared by gender.

Symptom	Females N = 1927	Males N = 1,321	*p* value*
Nausea	1.06(±1.45)	0.76 (±1.28)	0.001
Retching	0.75 (±1.26)	0.54 (±1.11)	0.001
Vomiting	0.39 (±0.95)	0.31 (±0.87)	0.02
Stomach fullness	1.26 (±1.52)	1.08 (±1.47)	0.001
Early satiety	1.23 (±1.50)	0.94 (±1.40)	0.001
Feeling excessively full after meals	1.13 (±1.53)	0.82 (±1.32)	0.001
Loss of appetite	1.07 (±1.47)	0.85 (±1.35)	0.001
Bloating	1.33 (±1.59)	0.99 (±1.44)	0.001
The stomach or belly is visibly larger	0.87 (±1.37)	0.63 (±1.17)	0.001

Values are expressed as mean ± standard deviation (±00). *p value is for a cross-group comparison; *post hoc* testing was conducted for significant findings.

A Binary Logistic regression analysis, shown in Table 4, was constructed to identify independent associated factors for suggestive gastroparesis. The model included sex, age, diabetes duration, HbA1c levels, and BMI. Binary regression indicated that the female sex increased symptom risk by 65% OR: 1.65% CI 1.35–2.01, *p*: 0.001. The model was significant (*p* < 0.0001) and correctly categorized 80.4% of the study participants as having at least one gastroparesis symptom.

**Table 4:** Logistic regression of any symptom suggestive of gastroparesis.

Factors	OR	95% CI	*p* value
BMI	1.00	0.992–1.000	0.943
Female sex	1.65	1.353–2.017	<0.001
Age	1.00	0.994–1.014	0.45
HbA1c	1.10	1.051–1.171	<0.0001
Disease duration	1.013	1.001–1.026	0.033

We performed binary logistic regression to identify independent associated factors for a severe picture of gastroparesis-related symptoms defined as GCSI: 3.5 or more. Covariants entered into the model as shown in Table 5. In this model, female gender was the only variable independently associated with a severe form of gastroparesis compared to male (OR: 2.38) *p* value (0.019) while no other studied variables showed a statistically significant association.

**Table 5:** Logistic regression of having a severe form of symptoms suggestive of gastroparesis.

Variables	OR	95% CI	*p* value
Age	0.973	0.941–1.006	0.106
Female sex	2.383	1.155–4.916	0.019
BMI	1.003	0.984–1.023	0.755
HbA1c category			0.855
HbA1c category (7–9)	1.210	0.499–2.935	0.674
HbA1c category (more than 9)	1.315	0.504–3.433	0.576
Diabetes duration			0.155
Duration (5–10)	1.070	0.380–3.018	0.898
Duration (more than 10)	2.065	0.777–5.484	0.146

## Discussion

Our study showed that females have a higher prevalence of all gastroparesis symptoms and more severe forms of symptoms when compared to males. This is consistent with previous studies that showed females were more susceptible to DGP than males (11, 17) and reported higher severity scores of DGP symptoms, especially nausea, early satiety, fullness, bloating, and loss of appetite (7, 18, 19). In the literature, 67–88% of DGP patients were females (20), with a prevalence that is 4 times higher in females according to meta-analysis and systematic review of published research (21). In the model assessing the presence of any gastroparesis-related symptoms, female sex, HbA1c levels, and diabetes duration were significantly associated. However, when focusing on severe symptom burden, only female sex remained independently associated, suggesting a stronger relationship between female sex and symptom severity rather than symptom presence alone.

The underlying mechanism is still controversial, several hypotheses may explain this observed association, however, given the cross-sectional design, these remain speculative and were not directly evaluated in the present study. One possible explanation is a slower gastric emptying in females potentially related to estrogen hormone and neuronal nitric oxide synthase that affects smooth muscle motility in the GI system (11, 12).

The nitrergic system, which consists of inhibitory enteric neurons, has also been suggested as a contributing factor. This system regulates gastric motility through nitric oxide (NO), an important inhibitory neurotransmitter in the gastrointestinal tract (11, 22). Some studies suggest that NO production may vary between males and females, which could partly explain differences in disease severity (23–25).

Gastric emptying may also be influenced by hormonal fluctuations across different physiological states. Variations in sex hormone levels during the menstrual cycle have been associated with changes in gastric motility, with the luteal phase—characterized by higher estrogen and progesterone levels—linked to slower gastric emptying and increased symptoms such as nausea and early satiety (13). A similar pattern has been observed during pregnancy, where persistently elevated hormone levels are associated with reduced gastric motility (17, 26, 27). Experimental studies, both *in vivo* and *in vitro*, further support the inhibitory effects of progesterone, with or without estrogen, on smooth muscle activity throughout the gastrointestinal tract (28–30) and hormonal changes in menopause and the use of exogenous hormones may also play a role (17).

Estrogen was also linked with NO through the regulation of nNOS. An observational study on rats’ gastric smooth muscle showed that an increased level of estrogen was associated with an increased level of NO, causing gastric muscle relaxation in female rats compared to males (31). Another study was done on FORKO female mice (mice with no estrogen, mimicking chronic estrogen deficiency) showed that chronic estrogen loss was associated with lower levels of NO (32), this study further implement that estrogen plays an important role in regulating NO and that low estrogen will cause impairment in the nitrergic system, negatively affects gastric motility (32), However, all research regarding this topic was done on mice and none on humans, which raises the need for further studies in this topic to confirm or alter suggested theories.

Beyond hormonal influences, some physiological differences make females more susceptible to DGP. For example, females have prolonged fundus relaxation, weaker antral contraction, and visceral hypersensitivity compared to males (33, 34). However, these observations are based on physiological and experimental studies and were not directly assessed in the present study.

Sex-related differences in symptom perception may also contribute to the observed findings. Previous studies suggest that females report gastrointestinal symptoms more frequently and may have higher visceral sensitivity and lower pain thresholds than males, which can influence symptom severity scores as a result, symptoms such as bloating, nausea, and early satiety may be reported more prominently in females (35). Moreover females tend to have a lower pain threshold and higher pain sensitivity that could influence severity of reported symptoms, this is due to multiple factors, including neurochemical, hormonal, genetic, psychological, and sociocultural (36, 37).

The relation between obesity and DGP is still controversial. Some research states that the classic presentation of DGP is a patient who is underweight and continues to lose weight (38), and other research considers it also a risk factor for DGP (39). In our research, obesity was not a risk factor for DGP nor its severity. Although BMI differed significantly between males and females, it did not remain an independent determinant of symptom severity after adjustment for other variables, suggesting that its crude association may be driven by confounding or interaction effects within the model. This pattern strengthens the interpretation that female sex itself is the primary factor linked to both the presence and increased severity of gastroparesis symptoms in this population.

DGP often happens after years of poorly controlled glycemic control in diabetic patients and has been linked with disease duration, where the duration of DM is considered a risk factor for DGP development. This could be explained as chronic DM is associated with autonomic neuropathy, which could be associated with DGP (1, 2).

HbA1c were also significantly associated with DGP, however, HbA1c>9 was not significantly associated with severity of DGP symptoms, this is similar to what we found in previous research that indicates that HbA1c level does not affect gastric motility (40), even though high HbA1c level is associated with increased odds of developing neuropathic complications of diabetes (41), taken together, these findings may suggest that factors other than glycemic control contribute to symptom severity in DGP; however, given the observational nature of this study, no conclusions can be drawn regarding the underlying pathophysiological mechanisms, including neuronal involvement.”

### Limitations

This study has several limitations. First, its cross-sectional design precludes establishing causal relationships between gender and gastroparesis symptoms. Second, the lack of longitudinal metabolic data limits our ability to assess temporal relationships between glycemic control and symptom progression. Additionally, several relevant confounders, including autonomic neuropathy, diabetic complications, medications affecting gastric motility, smoking, alcohol use, and socioeconomic factors, were not assessed and therefore not included in the regression models. This may have introduced residual confounding. Additionally, the assessment of gastroparesis was based on a symptom-based tool (GCSI) without confirmatory gastric emptying studies such as scintigraphy, which remains the gold standard for diagnosis. Therefore, our findings reflect symptom burden rather than a confirmed diagnosis of gastroparesis, and misclassification cannot be excluded. This reflects the limited availability of such diagnostic modalities in resource-limited settings like Palestine. Therefore, our findings represent symptom burden rather than a definitive diagnosis of gastroparesis. Additionally, data on symptoms and lifestyle factors were self-reported, which introduces the possibility of reporting bias. Participants were recruited from governmental hospitals, which may overrepresent patients with more advanced disease or specific socioeconomic backgrounds, thereby limiting the generalizability of the findings. Finally, although we adjusted for several variables, residual confounding cannot be excluded.

## Conclusion

The female gender is an associated factor for developing DGP and the only independent associated factor for disease severity. The underlying mechanism is not fully understood to this day. This raises the need for targeted research regarding this topic, as a better understanding of the pathogenesis of GDP and why it affects females more is a necessity to guide and implement gender-based management plans targeting factors that only exist in one gender. The best practice standards are to prevent or delay complications involving early diagnosis of diabetes and early intervention (42). Given the above, it is rational to pre-select diabetic patients for further evaluation and assessment based on the presence of cardinal symptoms related to gastroparesis.

## Data Availability

The original contributions presented in the study are included in the article/supplementary material, further inquiries can be directed to the corresponding author.
